# Epitope-based Multi-variant SARS-Cov-2 Vaccine Design: Shared Epitopes Among the Natural SARS-Cov-2 Spike Glycoprotein and 5 of its Variants (D614G, α, β, γ, δ) with High *in Silico* Binding Affinity to Human Leukocyte Antigen (HLA) Class II Molecules

**DOI:** 10.29245/2578-3009/2021/4.1223

**Published:** 2021-10-29

**Authors:** Spyros A. Charonis, Apostolos P. Georgopoulos

**Affiliations:** 1 The HLA SARS-CoV-2 Research Group, Brain Sciences Center, Department of Veterans Affairs Health Care System, Minneapolis, MN 55417, USA; 2 Department of Neuroscience, University of Minnesota Medical School, Minneapolis, MN 55455, USA

## Abstract and Introduction

The appearance and fast spread of five SARS-CoV-2 variants (D614G, B.1.1.7-UK [α], B.1.351-South Africa [β], P.1-Brazil [γ], B.167.2-India [δ]) have raised concerns regarding adaptive immunity, namely the extent to which antibodies against the original SARS-CoV-2 spike glycoprotein (S^natural^) would protect against those variants^1^. A related issue is how effective current vaccines are against the known variants of concern^2^. This issue is important because all current vaccines have S^natural^ as their target antigen. The first step in initiating antibody production is the formation of a complex between an epitope of the foreign antigen (here, a spike glycoprotein) and a Human Leukocyte Antigen (HLA) Class II molecule; this complex engages CD4+ T-lymphocytes for the initiation of antibody production by B cells (Major Histocompatibility Complex [MHC] restriction)^3–6^. Given the underlying mechanisms of long-term adaptive immunity, vaccines containing epitopes shared by all 6 spike glycoprotein variants and with high binding affinity to HLA Class II molecules could potentially be good candidates for offering a universal protection against SARS-Cov-2. Numerous efforts have been made in determining vaccine targets for SARS-CoV-2 using computational methods. These methods are based on B cell and T cell epitope prediction^7–14^ and indeed extend beyond SARS-CoV-2 to encompass prediction of the pathogen-based immune response more generically^15–19^. In the current study, we explored this approach by investigating *in silico* the binding affinities of all linear 15-, 18- and 22-amino acid long epitopes of S^natural^ and its 5 variants (S^D614G^, S^B.1.1.7^, S^B.1.351^, S^P.1^, S^P.167.2^) to 66 common HLA Class II alleles with global frequencies of ≥ 0.01. We identified 18 such epitopes which occur in all 6 spike glycoproteins and which bind with very high affinity to HLA Class II molecules. Most of these molecules came from the DPB1 gene. The suitability of these candidate epitopes for a successful multivariant SARS-CoV-2 vaccine design remains to be determined.

## Materials and Methods

The main objective of this study was to exhaustively assess the binding affinities of HLA Class II molecules to six variants of the SARS-CoV-2 spike glycoprotein. The variants and some of their sequence properties are summarized in Table 1. The point mutations and deletions of important SARS-CoV-2 variants are documented in several online repositories, with the CDC database (https://www.cdc.gov/coronavirus/2019-ncov/variants/variant-info.html) being used in this study.

## HLA Alleles

For this study, we selected the more frequent alleles of classical HLA Class II genes (DPB1, DQB1, DRB1), namely all alleles with frequencies ≥ 0.01, an arbitrary but reasonable threshold. For that purpose, we obtained an Estimation of Global Allele Frequencies by querying the relevant website^20^. The alleles with frequencies ≥ 0.01 that we used are listed in Table 2. They comprised 21, 15 and 30 alleles of DPB1, DQB1 and DRB1 genes, respectively.

## Partitioning the SARS-CoV-2 Spike Glycoprotein Variants

The amino acid sequences of five viral spike proteins (S^natural^, S^D614G^, S^B.1.1.7^, S^B.1.351^, and S ^P.1^) (Table 1) were retrieved from the UniprotKB database^21^. The amino acid sequence of the more recent Indian variant spike glycoprotein S^P.167.2^ was retrieved from the NCBI SARS-CoV-2 data hub^22^. The retrieved sequence (GenBank Acc: QWU05442.1) was matched by filtering the search for SARS-CoV-2 spike glycoprotein sequences from the B.167.2 lineage originating in India.

**Figure 1.**
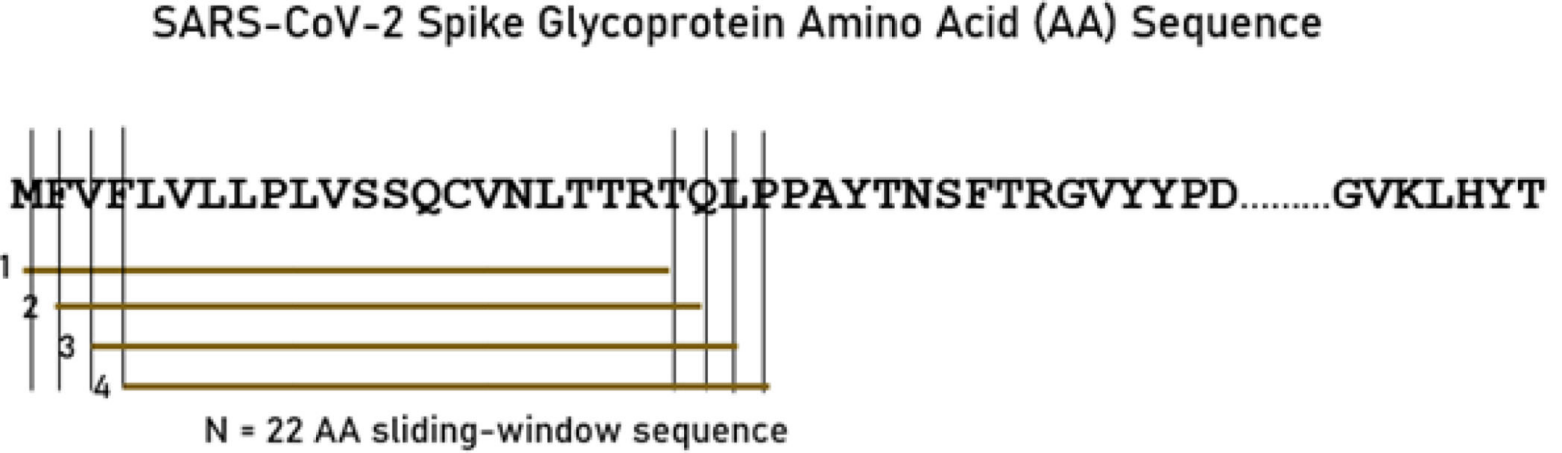
A sample of the sliding window approach^23^ for the spike glycoprotein variants (SARS-CoV-2 sequence displayed). See text for details.

Each viral sequence was queried for binding affinity against 66 common HLA Class II alleles (Table 2). A sliding epitope window approach^23^ was used to partition the sequence of the spike glycoprotein for each variant. Partitioning was done in a manner to obtain all possible consecutive linear 15-, 18- and 22-mers (e.g. for 15-mers residues 1–15, 2–16, …, *n*-15 where *n*=sequence length) that cover the entire sequence length (Fig 1). These peptide lengths are in the range of suitable lengths for binding with HLA Class II molecules^6^.

The partitioning was implemented in a Python script (version 3.8). All *n*-mers were queried in the IEDB database^24^ in order to determine their binding affinities to a set of 66 HLA Class II receptor molecules. Binding affinity predictions were obtained using the NetMHCIIpan method^25^. For each *n*-mer, a binding affinity score was predicted and reported as a percentile rank by comparing the peptide’s score against the scores of five million random *n*-mers selected from the UniProt database^21^. Smaller percentile ranks indicate higher binding affinity. For each gene locus (e.g. DRB1) and spike protein variant (e.g. Indian delta variant), all alleles and *n*-mers (formatted as a FASTA sequence alignment) were entered as a single query and, thus, the same set of 5 million random *n*-mers was employed to rank all queried alleles. Altogether, for each allele, 7544 15-mers, 7526 18-mers, and 7502 22-mers were tested for a total of 22572 n-mers x 66 alleles = 1498464 tests. Smaller percentile ranks indicate higher binding affinity; therefore, the lowest (i.e. minimum) percentile rank (LPR) for each allele (corresponding to the highest binding affinity) and *n*-mer of each spike glycoprotein was retrieved. Finally, we employed the most conservative threshold of LPR = 0.01 (the lowest LPR returned by NetMHCIIpan) to identify selected epitope sequences (*n*-mers) with LPR = 0.01 (highest affinity) for further analysis.

The locations in the primary sequence of all 15-, 18- and 22-mers with LPR = 0.01 were tabulated and the *n*-mers that overlapped with the receptor-binding domain (RBD) of the spike glycoprotein identified. This sequence-based quantity was calculated as the number of residues within the RBD (positions 338–506 in the linear sequence of the spike protein) divided by the length of the respective n-mer, i.e., 15/18/22. RBD proportion values were included because RBD is the structural region that binds to ACE2 receptors^26^. Indeed, serological studies of over 600 individuals infected with SARS-CoV-2 have shown that ~90% of the plasma or serum-neutralizing antibody activity targets the spike protein RBD^27^.

## Results

We identified 18 epitope sequences which occurred in all 6 spike glycoproteins and had HLA binding affinities of LPR = 0.01 for at least one allele. Table 3 shows the AA sequence of each epitope and their position in the relevant glycoprotein sequence. Table 4 shows the alleles for which a sequence had very high *in silico* binding affinity of LPR = 0.01, together with the sum of the population frequencies of those alleles as an estimate of global population coverage; this estimate will vary among different populations, depending on the allele frequency specific for a particular population. Finally, the fraction of overlap of each sequence with the RBD region is given in Table 5. Remarkably, all 4 sequences with most high binding affinities (#2, 3, 4, 12, in bold in Table 4) highly overlapped with the RBD region.

## Alleles Involved

Of the total 66 alleles tested (Table 2), 21 belonged to DPB1 gene, 15 to DQB1 gene, and 30 to DRB1 gene. On the other hand, there were 12 distinct alleles (Table 6) involved in high binding affinity with the 18 sequences identified. Most of them (8/12; 66.7%) belonged to the DPB1 gene, with only 2/12 coming from the DQB1 and DRB1 genes, each. With respect to the number of alleles tested, again the highest proportion of high affinity binding alleles came from the DPB1 gene (8/21; 38.1%), followed by the DQB1 gene (2/15; 13.3%) and the DRB1 gene (2/30; 6.7%).

## Discussion

The rationale of this study rests on the HLA restriction on antigen presentation to CD4+ T lymphocytes. More specifically, the formation of HLA Class II molecule-peptide antigen complex is a primary, necessary, although not sufficient, stage in successful antibody production, which depends on other factors downstream from CD4+ T cell activation. Here we focused on exhaustively screening *in silico* the affinity of linear continuous epitopes of the natural SARS-CoV-2 spike glycoprotein and of 5 of its variants (D614G, B.1.1.7, B.1.1351, P.1, P167.2) to common 66 HLA Class II molecules (global frequency ≥ 0.01) in an effort to identify epitopes occurring in all 6 spike glycoproteins that bind with very high affinity of HLA Class II molecules. We reasoned that such epitopes would be good candidates for vaccine(s) that would be effective against all 6 SARS-CoV-2 spike glycoproteins. Indeed, we identified 18 such epitopes that occur in all 6 glycoproteins and bind with very high affinity (LPR = 0.01) with various Class II alleles (Table 5). These epitopes pass the first screening for high affinity binding with HLA Class II molecules and, pending, further assessment^28–29^, are suitable candidates for being employed in a SARS-CoV-2 multivariant vaccine design. In addition, Table 4 provides estimates of global population coverage for each sequence with 4 sequences (#2, 3, 4, 12 in Table 4) offering high coverage; estimates of population coverage would vary for different populations, depending on the frequencies of the alleles involved in a particular population. Remarkably, the position of all those 4 sequences in each glycoprotein greatly overlaps with the RBD region.

Finally, with respect to the HLA Class II genes involved in this high affinity binding to epitopes of the 6 SARS-CoV-2 spike glycoproteins, the DPB1 gene provided the highest percentages of alleles involved (Table 6). In contrast, both the DQB1 and DRB1 genes were much less involved. The prominent involvement of the DPB1 gene is in keeping with our previous finding that the frequency of alleles of the DPB1 gene is positively associated with the binding affinity of epitopes of the SARS-CoV-2 natural spike glycoprotein^23^. This may reflect an evolutionary pressure favoring the selection of the DPB1 gene due to its presumed success in binding to coronaviruses, thus aiding survival and conferring evolutionary advantage.

## Limitations

The main limitation of these results is that they were derived using an *in silico* methodology. We believe that this approach is justified given the infeasibility of performing those binding affinity assessments experimentally.

## Figures and Tables

**Table 1.** SARS-CoV-2 spike glycoprotein variants.

Variant/Location	Nomenclature	Length of Viral Protein	N of 15-mers	N of 18-mers	N of 22-mers
Natural	SARS-CoV-2	1273	1258	1255	1251
D614G	D614G / Asp614Gly	1273	1258	1255	1251
UK (alpha, α)	B.1.1.7	1271	1256	1253	1249
South Africa (beta, β)	B.1.351	1273	1258	1255	1251
Brazil (gamma, γ)	P.1	1273	1258	1255	1251
India (delta, δ)	B.167.2	1271	1256	1253	1249

**Table 2.** HLA Class II alleles used, ordered by gene (color-coded) and their global frequencies in descending order. (See text for details.)

Index	Allele	Frequency
1	DPB1*04:01	0.23022
2	DPB1*101:01	0.17700
3	DPB1*05:01	0.16296
4	DPB1*04:02	0.16181
5	DPB1*02:01	0.15451
6	DPB1*03:01	0.06760
7	DPB1*01:01	0.05857
8	DPB1*13:01	0.04415
9	DPB1*14:01	0.04115
10	DPB1*02:02	0.02725
11	DPB1*09:01	0.02473
12	DPB1*17:01	0.02403
13	DPB1*28:01	0.01827
14	DPB1*77:01	0.01597
15	DPB1*11:01	0.01460
16	DPB1*18:01	0.01448
17	DPB1*107:01	0.01400
18	DPB1*10:01	0.01295
19	DPB1*21:01	0.01172
20	DPB1*22:01	0.01149
21	DPB1*06:01	0.01001
22	DQB1*03:01	0.24528
23	DQB1*02:01	0.13308
24	DQB1*03:02	0.10029
25	DQB1*05:01	0.09970
26	DQB1*06:02	0.07696
27	DQB1*02:02	0.07162
28	DQB1*03:03	0.05454
29	DQB1*04:02	0.05185
30	DQB1*06:01	0.05043
31	DQB1*05:02	0.04806
32	DQB1*05:03	0.04270
33	DQB1*06:03	0.04042
34	DQB1*06:04	0.02836
35	DQB1*04:01	0.02426
36	DQB1*06:09	0.01356
37	DRB1*07:01	0.11305
38	DRB1*15:01	0.09560
39	DRB1*03:01	0.08850
40	DRB1*11:01	0.07516
41	DRB1*01:01	0.06829
42	DRB1*13:01	0.05585
43	DRB1*11:04	0.05065
44	DRB1*04:01	0.04420
45	DRB1*13:02	0.03842
46	DRB1*16:01	0.03479
47	DRB1*14:01	0.03005
48	DRB1*14:54	0.02665
49	DRB1*15:02	0.02583
50	DRB1*12:01	0.02435
51	DRB1*04:04	0.02248
52	DRB1*09:01	0.02224
53	DRB1*04:05	0.02144
54	DRB1*08:01	0.02097
55	DRB1*12:02	0.02014
56	DRB1*04:03	0.01807
57	DRB1*01:02	0.01745
58	DRB1*13:03	0.01670
59	DRB1*04:11	0.01642
60	DRB1*08:03	0.01629
61	DRB1*04:07	0.01524
62	DRB1*16:02	0.01494
63	DRB1*14:02	0.01480
64	DRB1*10:01	0.01429
65	DRB1*08:02	0.01421
66	DRB1*04:02	0.01362

**Table 3.** Position of the 18 epitopes for S^natural^ and its 5 variants in Table 1.

			SARS-CoV-2 Variant					
Epitope	n-mer	AA Sequence	Natural	D614G	α	β	γ	δ
1	15	DEMIAQYTSALLAGT	867	867	864	866	867	865
2	15	FGEVFNATRFASVYA	338	338	336	338	338	336
3	15	GEVFNATRFASVYAW	339	339	337	339	339	337
4	15	PFGEVFNATRFASVY	337	337	335	337	337	335
5	15	QQLIRAAEIRASANL	1010	1010	1007	1009	1010	1008
6	15	TDEMIAQYTSALLAG	866	866	863	865	866	864
7	15	TQQLIRAAEIRASAN	1009	1009	1006	1008	1009	1007
8	18	FGEVFNATRFASVYAWNR	338	338	336	338	338	336
9	18	GEVFNATRFASVYAWNRK	339	339	337	339	339	336
10	18	LLTDEMIAQYTSALLAGT	864	864	861	863	864	336
11	18	LTDEMIAQYTSALLAGTI	865	865	862	864	865	337
12	18	PFGEVFNATRFASVYAWN	337	337	335	337	337	337
13	18	TYVTQQLIRAAEIRASAN	1006	1006	1003	1005	1006	863
14	22	ITNLCPFGEVFNATRFASVYAW	332	332	330	332	332	330
15	22	TNLCPFGEVFNATRFASVYAWN	333	333	331	333	333	331
16	22	NLCPFGEVFNATRFASVYAWNR	334	334	332	334	334	332
17	22	LCPFGEVFNATRFASVYAWNRK	335	335	333	335	335	333
18	22	CPFGEVFNATRFASVYAWNRKR	336	336	334	336	336	334

**Table 4.** HLA alleles for which a sequence had a very high in silico binding affinity of LPR = 0.01. The cumulative allele frequency is the sum of the global frequencies of the corresponding alleles, given in Table 2. The sequences with higher population coverage are in **bold**.

Epitope	n-mer	AA Sequence	Allele number (from Table 2)	Cumulative allele frequency
1	15	DEMIAQYTSALLAGT	38, 49	0.1214
2	15	FGEVFNATRFASVYA	1, 2, 4, 7, 10, 13, 14, 20	0.7006
**3**	15	GEVFNATRFASVYAW	1, 2, 4, 7, 10, 14, 20	0.5053
**4**	15	PFGEVFNATRFASVY	1, 4, 7, 14	**0.4666**
5	15	QQLIRAAEIRASANL	26, 30	0.1274
6	15	TDEMIAQYTSALLAG	38, 49	0.1214
7	15	TQQLIRAAEIRASAN	26, 30	0.1274
8	18	FGEVFNATRFASVYAWNR	1, 7, 20	0.3003
9	18	GEVFNATRFASVYAWNRK	20	0.0115
10	18	LLTDEMIAQYTSALLAGT	49	0.0258
11	18	LTDEMIAQYTSALLAGTI	49	0.0258
12	18	PFGEVFNATRFASVYAWN	1, 4, 7, 14	**0.4781**
13	18	TYVTQQLIRAAEIRASAN	30	0.0504
14	22	ITNLCPFGEVFNATRFASVYAW	1	0.2302
15	22	TNLCPFGEVFNATRFASVYAWN	1	0.2302
16	22	NLCPFGEVFNATRFASVYAWNR	1	0.2302
17	22	LCPFGEVFNATRFASVYAWNRK	1	0.2302
18	22	CPFGEVFNATRFASVYAWNRKR	1	0.2302

**Table 5.** RBD overlap of the 18 epitopes for S^natural^ and its 5 variants in Table 1.

			SARS-CoV-2 Variant					
Epitope	n-mer	AA Sequence	Natural	D614G	α	β	γ	δ
1	15	DEMIAQYTSALLAGT	0	0	0	0	0	0
2	15	FGEVFNATRFASVYA	0.93	0.93	0.80	0.93	0.93	0.80
3	15	GEVFNATRFASVYAW	1.00	1.00	0.87	1.00	1.00	0.87
4	15	PFGEVFNATRFASVY	0.87	0.87	0.73	0.87	0.87	0.73
5	15	QQLIRAAEIRASANL	0	0	0	0	0	0
6	15	TDEMIAQYTSALLAG	0	0	0	0	0	0
7	15	TQQLIRAAEIRASAN	0	0	0	0	0	0
8	18	FGEVFNATRFASVYAWNR	0.94	0.94	0.83	0.94	0.94	0.83
9	18	GEVFNATRFASVYAWNRK	1.00	1.00	0.89	1.00	1.00	0.83
10	18	LLTDEMIAQYTSALLAGT	0	0	0	0	0	0.83
11	18	LTDEMIAQYTSALLAGTI	0	0	0	0	0	0.89
12	18	PFGEVFNATRFASVYAWN	0.89	0	0.78	0.89	0.89	0.89
13	18	TYVTQQLIRAAEIRASAN	0	0	0	0	0	0
14	22	ITNLCPFGEVFNATRFASVYAW	0.68	0.68	0.59	0.68	0.68	0.59
15	22	TNLCPFGEVFNATRFASVYAWN	0.73	0.73	0.64	0.73	0.73	0.64
16	22	NLCPFGEVFNATRFASVYAWNR	0.77	0.77	0.68	0.77	0.77	0.68
17	22	LCPFGEVFNATRFASVYAWNRK	0.82	0.82	0.73	0.82	0.82	0.73
18	22	CPFGEVFNATRFASVYAWNRKR	0.86	0.86	0.77	0.86	0.86	0.77

**Table 6.** HLA alleles involved in very high in silico binding affinity of LPR = 0.01 with the 18 sequences.

Allele	Number of sequences involved	Percent of 18 sequences in which allele was involved
DPB1*01:01	5	27.8%
DPB1*02:02	2	11.1
DPB1*04:01	10	55.6
DPB1*04:02	4	22.2
DPB1*22:01	4	22.2
DPB1*28:01	1	5.6
DPB1*77:01	4	22.2
DPB1*101:01	2	11.1
DQB1*06:01	3	16.7
DQB1*06:02	2	11.1
DRB1*15:01	2	11.1
DRB1*15:02	4	22.2
